# Chronic Wasting Disease Exposure Risk to Male Cervids During the Mating Season in A Multi‐Host Community

**DOI:** 10.1002/ece3.74463

**Published:** 2026-10-09

**Authors:** Rebecca M. Windell, Jennifer L. Malmberg, Larissa L. Bailey, Scott Fesmire, Sydney Simpson, Marnee K. Roundtree, Georgia C. Titcomb

**Affiliations:** ^1^ Department of Fish, Wildlife, and Conservation Biology Colorado State University Fort Collins Colorado USA; ^2^ United States Department of Agriculture National Wildlife Research Center Fort Collins Colorado USA; ^3^ Nebraska Game and Parks Commission Wildlife Health Program Lincoln Nebraska USA

**Keywords:** cervid behavior, chronic wasting disease (CWD), exposure risk, local infectious zone, multi‐host disease

## Abstract

Multi‐host infectious diseases present myriad challenges to wildlife managers due to variation in spatial overlap, contact rates, and host competence that complicate understanding of exposure risk and transmission dynamics. These challenges are present in cervid systems impacted by chronic wasting disease (CWD), where CWD prevalence tends to be highest in male cervids. This study focuses on male cervids during the mating season in a multi‐host system where CWD is enzootic. We used wildlife camera traps placed at two types of potential local infectious zones—water tanks and scrape sites—and reference sites to measure spatial overlap and duration of contact behaviors for three susceptible cervid species: mule deer ( *Odocoileus hemionus* ); white‐tailed deer ( *Odocoileus virginianus* ); and elk ( *Cervus canadensis* ). For each species, we evaluated differences in intraspecific, interspecific, and total environmental exposure risk, along with direct exposure risk, at each site type. We also explored how varied assumptions of host competence across species affected interspecific exposure risk. For mule deer and white‐tailed deer, interspecific environmental exposure risk was 100 and 6 times higher at local infectious zones than at reference sites, and exclusively occurred at water tanks for elk. Interspecific environmental exposure risk occurred entirely at local infectious zones, but the proportion of total risk attributed to intraspecific vs. interspecific contributions depended greatly on whether we considered mule deer and elk to have equal host competence or used prevalence within a species as a proxy for variable host competence. Mule deer engaged in direct contact behaviors for similar durations across all site types, with no significant differences in direct exposure risk across sites. Our study highlights the potential central role of mule deer in multi‐host cervid communities impacted by diseases transmitted horizontally such as CWD, and provides insight for CWD surveillance and management during a period of particularly high male–male contact that is relevant to CWD epidemiology.

## Graphical abstract

Multi‐host cervid communities impacted by chronic wasting disease pose challenges for understanding exposure risk, especially during the mating season when male contact rates peak. Using camera traps at local infectious zones, we found that environmental interspecific exposure risk occurred almost exclusively at water tanks and scrape sites—reaching 100× higher for mule deer and 6× higher for white‐tailed deer relative to reference sites—and that mule deer played a central role in structuring both intra‐ and interspecific environmental exposure risk.

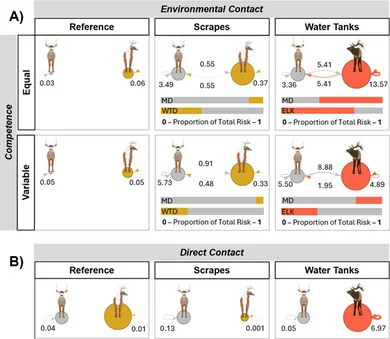

## 1 Introduction

Multi‐host pathogens account for most emerging diseases worldwide (Taylor et al. 2001), posing significant challenges for disease management in human, livestock, domestic animal, and wildlife populations. Complex wildlife disease dynamics often result in significant roadblocks as managers work to curb the frequency and spread of disease. Chronic wasting disease (CWD) – a fatal and transmissible prion disease affecting multiple cervid species including mule deer ( *Odocoileus hemionus* ), white‐tailed deer ( *Odocoileus virginianus* ), elk ( *Cervus canadensis* ), and moose ( *Alces alces* ) in North America—has presented wildlife managers with significant challenges given its complicated transmission dynamics, continued geographic spread, and potential impacts on cervid populations (Escobar et al. 2020).

CWD prions, the infectious agent of CWD, are shed by infected cervids in excretions and secretions (e.g., saliva, urine, and feces) and can remain infectious in the environment for multiple years (Denkers et al. 2024; Escobar et al. 2020; Henderson et al. 2015; Henderson et al. 2017; Potapov et al. 2013; Saunders, Bartelt‐Hunt, and Bartz 2012; Saunders, Bartz, and Bartelt‐Hunt 2012; Zabel and Ortega 2017). Transmission occurs horizontally via animal‐to‐animal contact (hereafter, direct contact) and via contact with contaminated environments and fomites (hereafter, environmental contact; Zabel and Ortega 2017). Social interactions (e.g., grooming, nose‐to‐nose contacts) and mating season activities (e.g., scraping, licking) provide opportunities for oral‐nasal transmission (Pritzkow 2022; Schauber et al. 2015). Direct contact likely drives much of the disease dynamics during the initial epizootic stage, whereas environmental and vertical transmission may become increasingly important as CWD becomes enzootic (Almberg et al. 2011; Miller et al. 2006; Sandoval et al. 2025).

Because sympatric cervid species infrequently come into direct contact, interspecific transmission is expected to occur primarily via environmental exposure in areas that accumulate prions over time and serve as prion reservoirs (i.e., local infectious zones; Blackburn et al. 2019; Lessler et al. 2017). Potential local infectious zones include areas with concentrated resources such as water and forage (e.g., water tanks, stored feed sites), along with areas where animals transmit social information (e.g., white‐tailed deer scrapes; Mejía‐Salazar et al. 2018). The probability of direct transmission, as well as the shedding and uptake of prions, likely varies across cervid species given differences in behaviors and resource selection. Here, behaviors associated with shedding, uptake, and direct transmission can serve as proxies for exposure risk.

Likelihood of both intra‐ and interspecific CWD transmission, as well as the potential for CWD emergence within a population, is further shaped by species‐specific susceptibility to CWD prions (Denkers et al. 2024; Haley et al. 2017, 2019; C. Johnson et al. 2006; C. J. Johnson et al. 2011; Kraft et al. 2023; Mysterud 2023; Plummer et al. 2017). CWD susceptibility varies across species and individuals to inform host competence—the ability of an infected host to transmit a pathogen to another susceptible host—resulting in differing levels of CWD prevalence. For example, mule deer and white‐tailed deer are both highly susceptible to CWD; however, in areas where both species occur, CWD prevalence in mule deer is higher (DeVivo et al. 2017; Miller et al. 2000; Mysterud 2023; Nobert et al. 2016; Rees et al. 2012; Smolko et al. 2021; but see Edmunds et al. 2016). This indicates that intraspecific transmission may be greater than interspecific transmission, and that transmission dynamics between these two species are only partially linked. In contrast, elk are less susceptible to CWD, and transmission dynamics appear to operate independently from mule deer and white‐tailed deer (Haley et al. 2017; Mysterud 2023). As a result, risk of intra‐ and interspecific CWD exposure is shaped by differences in host competence, along with the densities of infected and susceptible hosts, spatiotemporal patterns of habitat use and niche overlap amongst species, and frequency of behaviors that result in both direct and environmental transmission (Mysterud 2023; Storm et al. 2013).

CWD prevalence also varies between sexes; male cervids are typically 1.5–3 times more likely to be infected than females (Rogers et al. 2022). The exact mechanism for this variation is unknown; however, increased male‐to‐male contact during bachelor periods or the rutting season, direct contact with numerous does during polygamous mating activities, and behavioral susceptibility, such as increased contact with local infectious zones, have all been hypothesized (De Vos et al. 1967; Potapov et al. 2013, 2016; Rogers et al. 2022). For each of these proposed mechanisms, the mating season likely increases exposure risk. Therefore, increased understanding of male cervid use of potential local infectious zones during the mating season and how they behave at these sites can bolster understanding of CWD exposure risk and offer potential management solutions to minimize exposure risk during this period of altered behavior and increased contact.

Our objective was to understand how intra‐ and interspecific variation in male cervid spatial overlap and contact rates shape exposure risk at potential local infectious zones during the mating season on a mixed agricultural‐wildland landscape. For the purposes of this study, we defined exposure risk as the average duration that each cervid species in our study engaged in (1) oral‐nasal contact behaviors with the environment and (2) direct contact behaviors between conspecifics at two potential local infectious zones—water tanks and scrape sites—along with reference sites that provided background information on cervid contact rates across the broader landscape matrix. Our study provides a snapshot of how each cervid species in our study area contributes to and interacts with potential localized prion reservoirs during the mating season and shapes intra‐ and interspecific environmental exposure risk dynamics at these sites. The duration of species‐specific contact behaviors at each local infectious zone was recorded with wildlife camera traps and used to calculate resulting intraspecific, interspecific, and total environmental exposure risk, along with direct exposure risk, in a multi‐host system where CWD is enzootic. Lastly, we also explored how exposure risk may change under two assumptions: (1) equal host competence amongst all species (hereafter, “equal competence”) and (2) variable host competence across species, using published prevalence estimates as a proxy (hereafter, “variable competence”).

We tested two hypotheses in this study. First, we hypothesized that male cervid exposure risk during the mating season is spatially aggregated. Here, we predicted that both environmental and direct exposure risk would be higher at potential local infectious zones when compared to reference sites. Second, we hypothesized that environmental interspecific exposure risk would differ when assuming variable and equal host competence across species. Specifically, we predicted that mismatches in competence across species would increasingly alter our conclusions about interspecific environmental exposure risk as species overlap and differences in competence increased.

## 2 Methods

### 2.1 Study Area

Our study was conducted in the Wildcat Hills of western Nebraska's Panhandle, an approximately 9100 km^2^ area located entirely within the Nebraska Game and Parks Commission's (NGPC) Upper Platte Deer Management Unit (Figure 1). Approximately 97% of land in Nebraska is privately held (Heffelfinger and Krausman 2023), and the Wildcat Hills region holds some of the state's highest concentrations of public and conservation partner lands. Located south of Scottsbluff, Nebraska, USA, the Wildcat Hills include buttes, canyons, and ridges dominated by mixed ponderosa pine and juniper forest surrounded by short‐grass prairie and cultivated agricultural lands that primarily produce corn, dry edible beans, and sugar beets, along with hay, winter wheat, and alfalfa. The Wildcat Hills provide important habitat for resident mule deer, white‐tailed deer, elk, and bighorn sheep ( *Ovis canadensis* ) populations, along with cougars ( *Puma concolor* ), coyotes ( *Canis latrans* ), and bobcats ( *Lynx rufus* ). CWD was first detected in the Wildcat Hills in 2000 and is thought to be enzootic in mule deer and white‐tailed deer, but is unknown for elk in this region. Beginning in 2015, NGPC has estimated CWD prevalence on a rotating basis across five districts, primarily using mature bucks (2.5 years and older) harvested during an annual nine‐day firearm season (NGPC 2024). CWD prevalence in mule deer (predominantly bucks) was estimated to be ~15% in the Upper Platte Deer Management Unit—the unit in which the Wildcat Hills is located—over the past decade and is unknown for white‐tailed deer and elk in this management unit (NGPC 2024, unpublished data).

**FIGURE 1:**
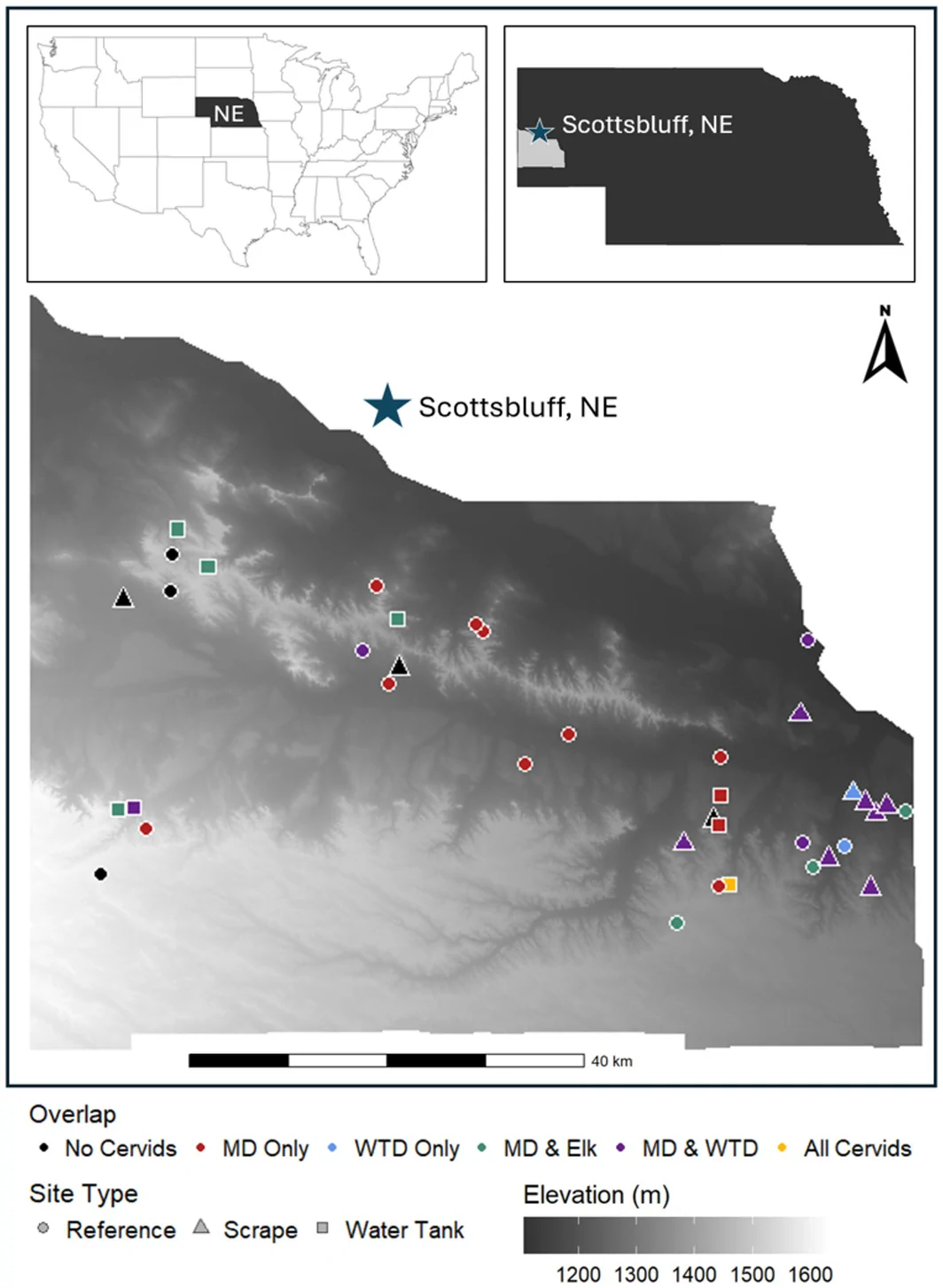
Spatial overlap amongst three male cervid species – mule deer (MD), white‐tailed deer (WTD), and elk—at camera locations in the Wildcat Hills near Scottsbluff, Nebraska (NE), USA. Cameras were set at three different site types—reference sites (*n* = 19, circles), white‐tailed deer scrape sites (*n* = 11, triangles), and water tanks (*n* = 8, squares)—to capture patterns of overlap and behaviors relevant to environmental and direct CWD exposure during the breeding season (16 October 2023 and 7 December 2023).

### 2.2 Camera Deployment

We opportunistically deployed 38 wildlife camera traps at three site types during the cervid mating season (Figure 1). Monitored site types included two potential local infectious zones—water tanks (*n* = 8) and white‐tailed deer scrape sites (*n* = 11)—and reference sites (*n* = 19). Water tanks were large steel stock tanks used by both cattle and wildlife, scrape sites were identified by the presence of overhanging branches and bare patches of soil commonly used by male white‐tailed deer during the mating season, and reference sites comprised areas of expected or known background deer activity (e.g., game trails, edge habitat along agriculture areas). Camera traps were active between 16 October 2023 and 7 December 2023, programmed to take 10 s videos with no delay when motion was detected, and each camera trap sampled an approximately 120 m^2^ area.

### 2.3 Duration of Contact Behaviors and Spatial Overlap

We tested our hypotheses regarding differences in intra‐ and interspecific environmental exposure risk across site types by quantifying both the mean daily density (sec) of contact behaviors relevant to environmental or direct CWD exposure risk and spatial overlap among male cervid species at each camera location.

#### 2.3.1 Contact Behaviors

We constructed an ethogram of contact behaviors, defining environmental contact behaviors as those that would result in both the uptake and shedding of prions, and direct contact behaviors as those that captured or preceded direct contact between a male cervid and a male or female of the same species (Table 1). Most environmental contact behaviors (i.e., investigating, drinking, foraging, scraping/thrashing) have the potential to result in both the shedding and uptake of prions; however, a notable exception are excretion behaviors (urinating/defecating) that only shed prions into the environment (Table 1). We only documented excretion behaviors in white‐tailed deer at scrape sites for < 60 s and consequently assumed all other environmental behaviors would result in both the uptake and shedding of prions for exposure risk calculations (see *Exposure Risk Analysis* below).

**TABLE 1:** Ethogram of contact behaviors exhibited by male cervids recorded by wildlife camera traps. Behaviors were categorized by chronic wasting disease exposure risk mode (direct or environmental). Direct contact behaviors documented or preceded direct contact between a male cervid and a male or female of the same species. Environmental contact behaviors contributed to indirect, environmental shedding, and uptake of prions.

Category	Behavior	Description
Direct	Courting	Male chasing female (no contact) or contacting body of female
Direct	Sparring/Fighting	Antler to antler contact
Environmental	Excretion*	Actively depositing scat or urine
Environmental	Investigating	Slowly moving/stationary with head down with evidence of ground contact OR contacting other surfaces (e.g., water tank edge, camera)
Environmental	Foraging	Slowly moving or stationary with head down, nose/mouth contacting ground, and/or chewing/moving lips, or head down for extended periods
Environmental	Drinking	Animal contacting water or seen with water dripping from mouth after head in water source, can hear drinking
Environmental	Scraping/Thrashing	Hoof scrape, antler, or tongue in contact in localized area of vegetation; associated with male scent marking/sign‐post behavior

* Shedding only.

Next, we applied our ethogram to all camera trap videos using Timelapse2 (Greenberg et al. 2019) by first coding videos to species and sex. We only included videos containing antlered male mule deer, white‐tailed deer, and elk. All video clips containing male cervids were consolidated into 1‐min events and coded to extract the contact behaviors of each male cervid. Contact behaviors observed in each event were classified as having environmental or direct exposure risk potential (Table 1) and summed for each camera location (site). Finally, site‐specific mean daily duration (sec) of contacts for each exposure risk type was calculated by scaling the total number of events to account for camera malfunctions that yielded variable video lengths (mean: 8.19 s; range: 1–60 s) and the number of days each camera was operational (mean: 36.4 days; range: 4.2–51.1 days).

Finally, cervid density is a critical factor in understanding potential CWD exposure risk (Mysterud 2023; Mysterud et al. 2023). Potential for CWD exposure is thus considered the product of (1) prion density in the environment (assumed constant given inability to quantify), (2) the rate of shedding and uptake behaviors, and (3) the density of hosts. Consequently, the density of contact behaviors within each camera's 120m^2^ viewshed are the product of the rate of shedding and uptake behaviors, and mean count of individuals engaged in these behaviors. Or in other words, the density of contact behaviors is the sum of the contact behaviors of all individuals at each camera location calculated as individual seconds of contacts per 120m^2^ (Titcomb et al. 2021, 2023).

#### 2.3.2 Spatial Overlap

Spatial overlap was determined by cooccurrence of two or more species at a given camera location. Probability of overlap between two or more species at each site type was estimated using a multinomial logistic regression model implemented in the *nnet* package (Venables and Ripley 2002) and used to compare the change in overlap odds between reference and potential local infectious zone sites for observed species pairs.

### 2.4 Host Competence

We evaluated the relative contribution of predicted interspecific environmental exposure risk between species pairs under assumptions of equal and variable host competence, using differences in CWD prevalence amongst sympatric mule deer, white‐tailed deer, and elk as a proxy for variable competence (Titcomb et al. 2021). CWD prevalence for mule deer, white‐tailed deer, and elk have not been consistently collected in the Wildcat Hills, although CWD prevalence in mule deer was most recently estimated at approximately 15% (2017, 2022, NGPC unpublished data). Hence, we conducted a systematic literature review to estimate interspecific variation in CWD prevalence across sympatric mule deer, white‐tailed deer, and elk. To conduct the literature search we used the Boolean search string: “chronic wasting disease” AND “mule deer” AND “white‐tailed deer” AND “elk” AND “prevalence” in Web of Science on March 23rd, 2025. We included all studies (1) in which CWD prevalence was calculated or estimated for free‐ranging mule deer, white‐tailed deer, and elk in the same study region; (2) in an area where CWD had been present for at least 1 year; and (3) where CWD prevalence was greater than 1% in any of the three species to ensure reasonable statistical power to estimate interspecific variation in prevalence. The search returned 52 studies, of which only two studies met our inclusion criteria (Table 2). Both studies occurred near the Wildcat Hills and documented CWD dynamics in similar enzootic systems; hence, the average prevalence values from these studies were used to approximate species‐specific competence values (i.e., variable competence), with respect to estimated mule deer CWD prevalence, in the Wildcat Hills (mule deer = 15%, white‐tailed deer = 8%, elk = 3.3%). Equal competence was estimated at a mean prevalence value of 9.15%.

**TABLE 2:** Chronic wasting disease prevalence was reported in two studies of sympatric mule deer (MD), white‐tailed deer (WTD), and elk (ELK; Miller et al. 2000, DeVivo et al. 2017) where prevalence was a minimum of 1% for at least one study species. The average prevalence between these two studies was used to approximate differences in variable and equal host competence for models of interspecific environmental exposure risk dynamics in Nebraska's Wildcat Hills. While baseline prevalence was substantially different (5–10 fold) across the Miller et al. 2000 and DeVivo et al. 2017 studies, the prevalence ratios between species (MD:WTD and MD:ELK) were more similar (1.3–2 fold differences).

References	Location	Years	MD	WTD	ELK	MD:WTD	MD:ELK
DeVivo et al. 2017	Wyoming, USA	2010–2014	24%	13%	6%	1.8	4.1
Miller et al. 2000	Colorado & Wyoming, USA	1996–1999	5%	2%	1%	2.3	9.8
	Wildcat Hills, Nebraska, USA	*Average*	*14%*	*8%*	*3%*	*1.9*	*4.5*
		Estimated^*^/Assumed†	15%*	8%†	3.3%†	1.9†	4.5†

### 2.5 Exposure Risk Analysis

We used estimated mean daily duration of contact behaviors to calculate species‐specific direct and environmental exposure risk at each site type (Titcomb et al. 2021, 2023). Environmental exposure to CWD was calculated in terms of intraspecific (*E*_*intra*_), interspecific (*E*_*inter*_), and total (*E*_*total*_) risk, where total environmental exposure risk (*E*_*total*_) was calculated as a combination of intraspecific (*E*_*intra*_) and interspecific (*E*_*inter*_) risk. Intraspecific environmental exposure risk was calculated for each species at sites where a species occurred, whereas interspecific environmental exposure risk was only calculated at sites where different cervid species overlapped. Direct CWD exposure risk was only estimated intraspecifically as we did not observe any direct interspecific contacts. To test hypotheses of exposure risk variation in response to differences in host competence, we calculated environmental exposure risk behaviors separately considering both equal and variable host competence (see *Host competence* above).

In calculating exposure risk (formulas outlined below), we highlight several key assumptions: (1) all environmental contact behaviors were assumed to result in equal amounts of prions shed and ingested; (2) daily time engaged in environmental contact behaviors resulted in the equal potential for each species to shed and ingest prions; (3) female cervid densities were constant across all sites; (4) spatial overlap among species at sites reflected habitat preferences, competition effects, and niche overlap; (5) the same proportion of susceptible and infected individuals is found at all sites; (6) prion persistence and bioavailability in the environment are the same at all sites; and (7) host competence is proportional to prevalence (in both equal and variable scenarios) and does not vary by individual within a species.

Intraspecific environmental exposure risk for members of each cervid species (*j*) was estimated at each site (*i*) as the product of uptake behaviors (*U*), host competence (*C*; Table 2), and shedding behaviors (*S*) as,

$$E_{{\text{intra}}_{\mathrm{ij}}}=U_{\mathrm{ij}}*C_{j}*S_{\mathrm{ij}}$$

where uptake and shedding behaviors were drawn from all behaviors associated with environmental contact by male cervids (Table 1). Interspecific environmental exposure risk was similarly estimated for cervid species (*j*) from each sympatric species (*k*) detected at site (*i*):

$$E_{{\text{inter}}_{\mathrm{ijk}}}=U_{\mathrm{ij}}*C_{k}*S_{\mathrm{ik}}$$

Total environmental exposure risk for species (*j*) was estimated as the sum of intra‐ and interspecific exposure risk from each sympatric cervid species (*n*) detected at site (*i*):

$$E_{{\text{total}}_{\mathrm{ij}}}=E_{{\text{intra}}_{\mathrm{ij}}}+\sum_{k=1}^{n}E_{{\text{inter}}_{\mathrm{ijk}}}$$

To account for potential effects of female shedding on male exposure risk, we modified *S*_*ij*_ and *S*_*ik*_ from the equations above to include an added female contribution to intraspecific environmental exposure as:

$$E_{{\text{intra}}_{\mathrm{ij}}}=U_{\mathrm{ij}}*C_{j}*\left(S_{\mathrm{ij}}+\left(F_{\mathrm{lj}}*S_{\mathrm{ij}}\right)\right)$$

And interspecific environmental exposure as:

$$E_{{\text{inter}}_{\mathrm{ijk}}}=U_{\mathrm{ij}}*C_{k}*\left(S_{\mathrm{ik}}+\left(F_{\mathrm{lk}}*S_{\mathrm{ik}}\right)\right)$$

Female contribution to shedding was calculated by weighting male shedding behaviors for each species and site by the ratio of observed females to males (*F*) at each site type (*l*). Female to male ratios were 3.83, 1.24, and 4.93 at reference, scrape, and water tanks sites for mule deer, and 1.45 and 2.06 at reference and scrape sites for white‐tailed deer. By weighting shedding behaviors as such, environmental exposure risk calculations including female contributions provide an upper bound of environmental exposure risk potential to males for each species and site type. In contrast, calculations not including the contribution of female shedding behaviors conservatively estimated risk to male cervids.

Exposure risk resulting from direct, intraspecific contacts was estimated at each site for each species. When two animals directly contacted each other, the potential for CWD exposure could arise from either individual. Hence, we calculated direct exposure risk as a product of the direct contact behavior (*X*; Table 1) exhibited by each individual involved in the contact and host competence:

$$E_{{\text{direct}}_{\mathrm{ij}}}={X_{\mathrm{ij}}}^{2}*C_{j}$$

Following calculation of site‐specific mean daily exposure risk for each species (*j*), exposure type, and competence scenario, we fit a set of Tweedie distributed generalized linear models (GLMs) using the *glmmTMB* package (Brooks et al. 2017). We used GLMs with Tweedie distributed errors to reflect the continuous and positive nature of our data with a point mass at zero. Models examining the relationship between risk and site type were fit to estimate risk for each species (3 species: mule deer, white‐tailed deer, elk) and exposure risk type (4 types: intraspecific, interspecific, and total environmental exposure risk, along with direct exposure risk). For environmental risk models, we considered two host competence scenarios (equal in which all species had the same competence; and variable, in which different species had competence informed by the literature (Table 2)). This resulted in a total of 18 environmental exposure risk models (3 species × 3 exposure risk types × 2 competence scenarios) and 2 direct exposure risk models (2 species; not enough data available for elk). We also fit all environmental risk models using calculations that accounted for the added contribution of female shedding (18 additional models). Separate models were fit to achieve stable coefficients, to address convergence issues resulting from missing data, and to ease interpretation. We used the *DHARMa* package (Hartig 2024) to check model assumptions and models were evaluated for statistical clarity (Dushoff et al. 2019). We extracted the estimated marginal means from each model and pairwise contrasts using the *emmeans* package (Lenth 2025).

Finally, we used network graphs to visualize how exposure risk is shaped by both the density of contact behaviors and spatial overlap between species (Craft 2015), including the relative contributions of intra‐ and interspecific exposure risk to total environmental risk using the *igraph* package (Csardi 2014). In our exposure risk networks, nodes represented relative exposure risk for each species at each site type, directed edges between nodes represented exposure risk from one species to another, and self‐loops at the nodes displayed the weight of intraspecific exposure risk (Farine and Whitehead 2015). We also estimated the relative contributions of intra‐ vs. interspecific exposure to total environmental exposure risk with respect to variable and equal host competence at each site type. All analyses were conducted in Program R v.4.4.1 (R Core Team 2024).

## 3 Results

### 3.1 Duration of Contact Behaviors and Spatial Overlap

Camera traps captured a total of 1837 videos of male cervids over the duration of the study, which collectively recorded 96.8 and 22.9 min of male cervid environmental exposure and direct exposure behaviors, respectively (Figure 2). Male mule deer engaged in both environmental and direct contact behaviors at each of the monitored site types, whereas we only observed white‐tailed deer engaged in contact behaviors at reference and scrape sites, and elk only engaged in contact behaviors at water tanks.

**FIGURE 2:**
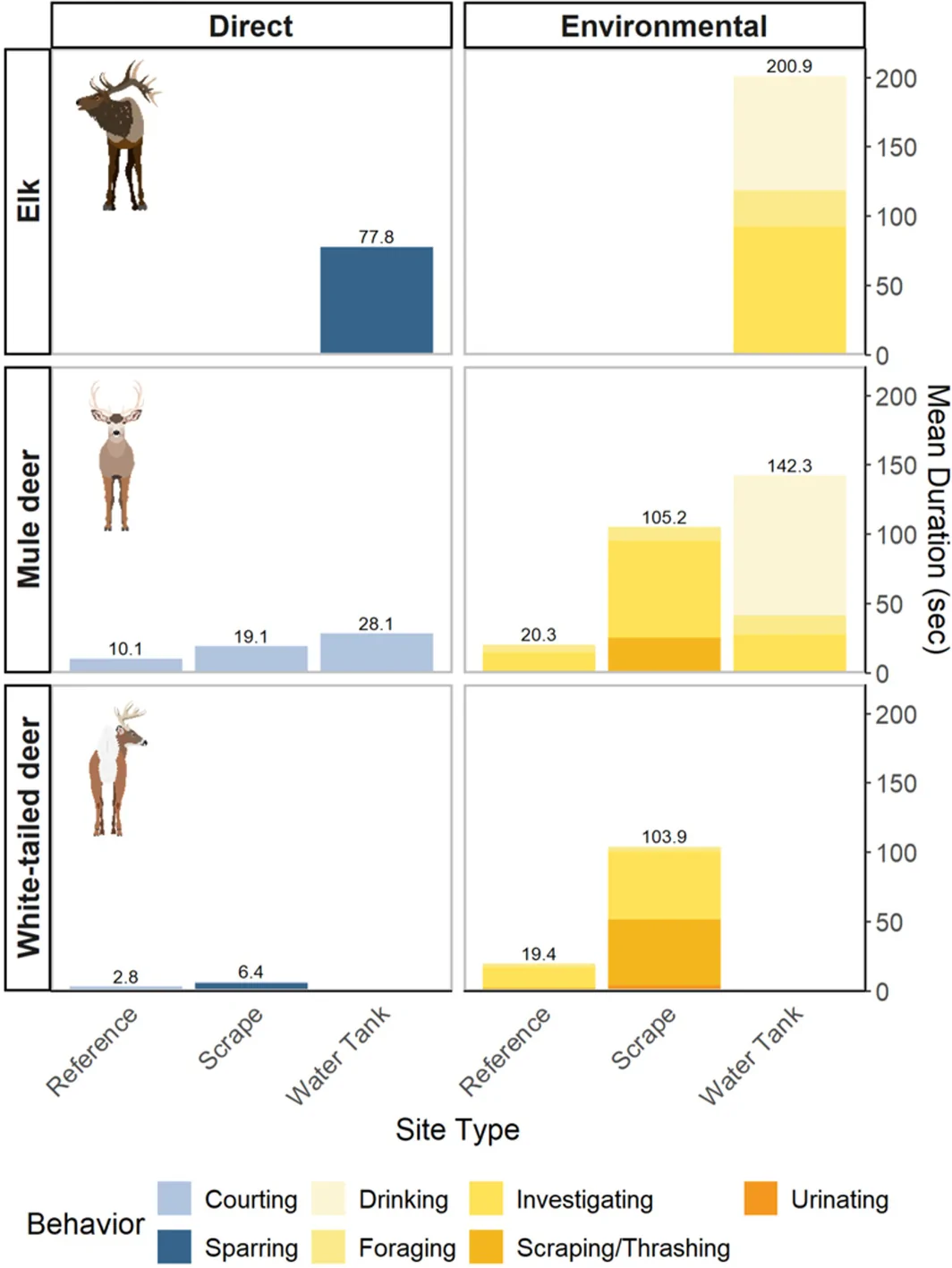
Observed mean durations (sec) of direct and environmental contact behaviors displayed by male cerivds during the mating season at reference (*n* = 19), scrape (*n* = 11), and water tank (*n* = 8) site types as documented by camera trap video footage. Elk, mule deer, and white‐tailed deer engaged in a total of 10.4, 10.5, and 2.1 min of direct contact behaviors, respectively, including courting and sparring activites. Enviromental contact behaviors resulting in potential CWD shedding and uptake included drinking, foraging, investigating, scraping/thrashing, and urinatation. Elk, mule deer, and white‐tailed deer engaged in a total of 26.8, 44.7, and 25.1 min, respectively, of environmental exposure behaviors. Mean durations (sec) of contact behaviors for each site type and species are printed above each bar. Species‐specific mean *daily* durations of behaviors at each camera location were used in calculations of direct and environmental exposure risk.

*Contact behaviors*: The specific environmental and direct contact behaviors male cervids engaged in varied by species and site type (Figure 2). Mean duration of environmental contact behaviors for mule deer at local infectious zones was 142 and 105 s at water tanks and scrape sites, compared to 20 s at reference sites. For white‐tailed deer, mean duration of environmental contact behaviors was 104 s at scrape sites and 19 s at reference sites. Elk engaged in a mean of 201 s of environmental contact behaviors exclusively at water tanks. While mule deer and white‐tailed deer spent similar amounts of time at scrape sites, they engaged in different scent marking behaviors, with white‐tailed deer interacting with scrape patches and overhanging lick branches, and mule deer thrashing their antlers on surrounding small trees and shrubbery (Figure 2).

For direct contact behaviors, mule deer engaged exclusively in courting behaviors for mean durations of 26 s, 19 s, and 10 s at water tank, scrape, and reference sites, respectively. Elk engaged in a mean duration of 78 s of sparring behaviors at water tanks; however, these observations were concentrated at only two water tank sites. Sparring behavior between male white‐tailed deer was observed for a mean duration of 3 s at reference sites and 6 s at a single scrape site. Additionally, we did not observe environmental or direct contact behaviors for elk at reference sites and white‐tailed deer at water tanks, despite both species being present in these locations (Figure 2).

#### 3.1.1 Spatial Overlap

Mule deer were the only species that overlaped with both white‐tailed deer and elk at multiple site types, with the highest probability of overlap between mule deer‐white‐tailed deer and mule deer‐elk occuring at scrape sites (0.64) and water tanks (0.50), respectively (Figure 3). In comparison to reference sites, the odds of mule deer and white‐tailed deer overlaping at scrape sites was 7.6 times higher ($\hat{\beta }$_MD‐WTD,Scrape_ = 2.03, SD = 0.90), and the odds of mule deer and elk overlaping at water tanks was 8.7 times higher ($\hat{\beta }$_MD‐ELK,Water_ = 2.16,SD = 1.08). All three cervid species overlapped at one water tank site and mule deer and white‐tailed deer overlapped at an additional water tank site; however, white‐tailed deer were not observed engaging in shedding or uptake behaviors at these sites (Figure 3).

**FIGURE 3:**
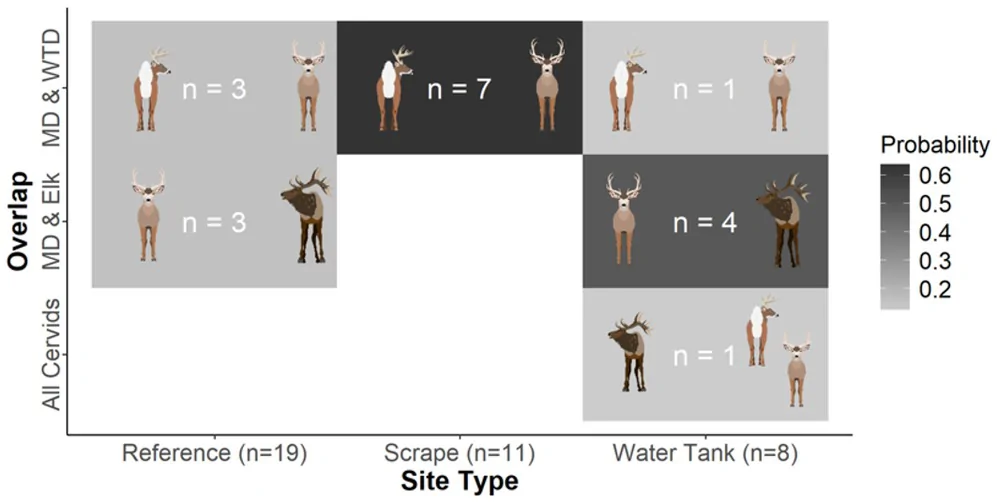
Probability of overlap and number of sites (*n*) where multiple cervid species overlapped across two potential local infectious zones (LIZs)—scrape sites and water tanks—and reference sites. Mule deer were the only species that overlaped with both white‐tailed deer and elk at multiple site types, with the highest probability of overlap between mule deer‐white‐tailed deer and mule deer‐elk occuring at scrape sites (0.64) and water tanks (0.50), respectively. All three cervid species overlapped at one water tank site; however, white‐tailed deer where not observed engaging in shedding or uptake behaviors at this site.

### 3.2 Exposure Risk Analysis

Total environmental exposure risk was highest at local infectious zones where multiple species overlapped; however, we found no clear differences in direct exposure risk with respect to site type for mule deer, the only species for which we had enough data to analyze differences in direct contact behaviors across site types. Pairwise contrasts of environmental risk between site types remained largely unchanged when weighted by the additional contribution of females (Table 4).

#### 3.2.1 Environmental Exposure Risk

We found statistically clear differences in environmental exposure risk across sites for intraspecific and total mule deer and white‐tailed deer exposures (Figure 4A, Table 3). Specifically, mule deer intraspecific and total exposure risk was more than 100 times higher at water tanks and scrape sites compared to reference sites, whereas white‐tailed deer exposure risk at scrape sites was 6 and 15 times higher for intraspecific and total environmental risk than at reference sites (Table 4).

**FIGURE 4:**
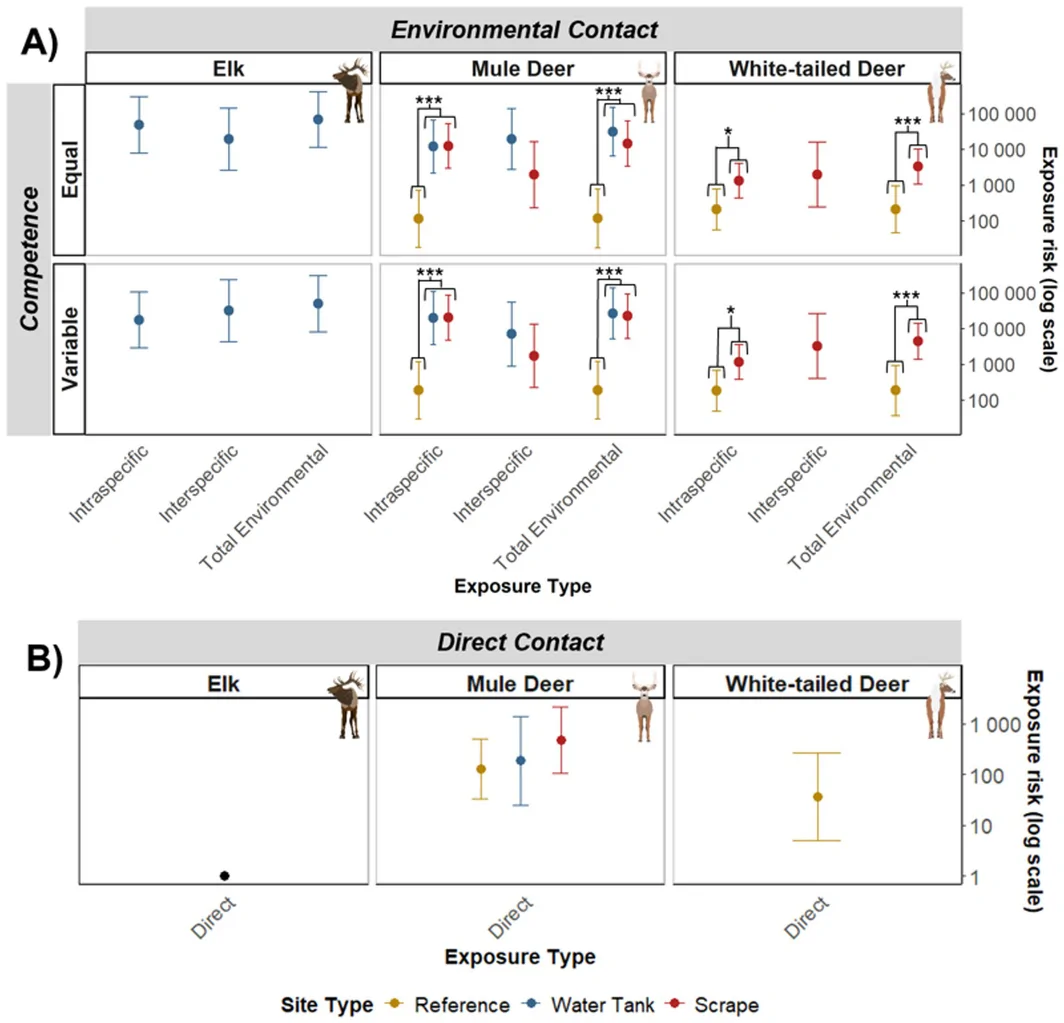
Log‐transformed estimated marginal means from generalized linear models of (A) intraspecific, interspecific, and total environmental exposure risk, and (B) direct exposure risk for elk, mule deer, and white‐tailed deer in response to site type with 95% confidence intervals. Site types included reference sites (yellow), water tanks (blue), and white‐tailed deer scrape sites (red). No estimate of direct exposure risk was available for elk (black). Asterisks denote models with statistically clear differences in exposure risk type across different site types at *p* < 0.001 (***) and *p* < 0.05 (*). For environmental contact networks we compared differences in intra‐ and interspecific exposure risk when considering both equal competence across all species (prevalence = 9.15%) and variable competence for sympatric mule deer (prevalence = 15%), white‐tailed deer (prevalence = 8%), and elk (prevalence = 3.3%).

**TABLE 3:** Estimated effects of site type and associated standard errors (in parentheses) from Tweedie distributed generalized linear models of calculated intraspecific (*E*_*intra*_), interspecific (*E*_*inter*_), and total (*E*_*total*_) exposure risk to mule deer, white‐tailed deer, and elk. The reference level site type varied by model; intercept values are presented in blue. Estimates in **bold gray** and **bold black** are statistically clear at *p* < 0.05 and *p* < 0.001, respectively.

			Exposure type			
Species	Competence	Term	*Intraspecific*	*Interspecific*	*Total enviro*.	*Direct*
Mule deer	*Equal*	Reference	4.744 (0.940)	—	4.769 (0.971)	4.861 (0.697)
		Scrape/rub	**4.695 (1.195)**	7.595 (1.087)	**4.817 (1.224)**	1.298 (1.035)
		Water tank	**4.655 (1.280)**	2.282 (1.478)	**5.591 (1.25**8)	0.364 (1.236)
	*Variable*	Reference	5.239 (0.940)	—	5.252 (0.947)	—
		Scrape/rub	**4.695 (1.195)**	7.461 (1.037)	**4.763 (1.193)**	—
		Water tank	**4.655 (1.280)**	1.397 (1.475)	**4.946 (1.261)**	—
White‐tailed deer	*Equal*	Reference	5.343 (0.671)	—	5.234 (0.773)	3.599 (1.011)
		Scrape/rub	**1.859 (0.879)**	7.595 (1.070)	**2.754 (0.963)**	—
	*Variable*	Reference	5.209 (0.671)	—	5.234 (0.826)	—
		Scrape/rub	**1.859 (0.879)**	8.090 (1.070)	**3.163 (1.017)**	—
Elk	*Equal*	Water tank	10.796 (0.921)	9.878 (1.024)	11.132 (0.913)	—
	*Variable*	Water tank	9.776 (0.921)	10.372 (1.024)	10.811 (0.925)	—

**TABLE 4:** Pairwise contrasts of estimated marginal CWD exposure risk means between site types (reference (R), scrape (S), and water tank (W)) for each competence scenario, species, and environmental exposure type, when considering risks resulting solely from male contacts (left) and risk from male contacts that include a female contribution to shedding (right). Risk is reported relative to the site in the denominator of each pairwise contrast, with associated standard error (SE), z‐ratio, and *p*‐value. The significance of pairwise exposure risk contrasts between site types remains unchanged when evaluated at *p* < 0.05 when considering only male contacts (lower bound of risk) and male contacts with an added female contribution to shedding (upper bound of risk).

Competence	Species	Exposure type	Pairwise contrast	Risk from male contacts only				Risk from male contacts with female contribution			
				Risk	SE	z‐ratio	*p*‐value	Risk	SE	z‐ratio	*p*‐value
*Variable*	Mule deer	Intraspecific	S/R	109.41	0.01	−3.93	**< 0.001**	50.75	0.02	−3.33	**0.003**
			W/R	105.11	0.01	−3.64	**0.001**	129.01	0.01	−4.03	**< 0.001**
			W/S	0.96	1.19	0.04	0.999	2.54	0.43	−0.85	0.672
		Interspecific	W/S	4.04	0.36	−0.95	0.344	1.38	1.09	−0.21	0.831
		Total	S/R	117.09	0.01	−3.99	**< 0.001**	56.21	0.02	−3.44	**0.002**
			W/R	140.56	0.01	−3.92	**< 0.001**	136.15	0.01	−4.10	**< 0.001**
			W/S	1.20	0.92	−0.17	0.985	2.42	0.44	−0.82	0.689
	White‐tailed deer	Intraspecific	S/R	6.42	0.14	−2.11	**0.035**	8.00	0.11	−2.35	**0.019**
		Total	S/R	23.64	0.04	−3.11	**0.002**	23.11	0.04	−3.15	**0.002**
*Equal*	Mule deer	Intraspecific	S/R	109.41	0.01	−3.93	**< 0.001**	50.75	0.02	−3.33	**0.003**
			W/R	105.11	0.01	−3.64	**0.001**	129.01	0.01	−4.03	**< 0.001**
			W/S	0.96	1.19	0.04	0.999	2.54	0.43	−0.85	0.672
		Interspecific	W/S	9.80	0.15	−1.54	0.123	3.34	0.44	−0.82	0.414
		Total	S/R	123.65	0.01	−3.94	**< 0.001**	60.94	0.02	−3.49	**0.001**
			W/R	268.06	0.00	−4.44	**< 0.001**	163.58	0.01	−4.26	**< 0.001**
			W/S	2.17	0.50	−0.71	0.759	2.68	0.40	−0.93	0.623
	White‐tailed deer	Intraspecific	S/R	6.42	0.14	−2.11	**0.035**	8.00	0.11	−2.35	**0.019**
		Total	S/R	15.71	0.06	−2.86	**0.004**	16.24	0.06	−2.94	**0.003**

We were unable to estimate differences in interspecific environmental exposure risk between reference and potential local infectious zone sites for any of our cervid species due to the low or non‐existent occurrence of interspecific contacts with the environment at reference sites. Mule deer and white‐tailed deer overlapped at three reference sites (Figure 3); however, very small durations (~1 s) of environmental contact behaviors exhibited by each species at a single site precluded estimation. This limited our ability to test the prediction that interspecific exposure risk was greater at local infectious zones, but emphasizes that exposure risk between mule deer and white‐tailed deer effectively only occurs at local infectious zones, such as scrapes. Similarly, while mule deer and elk overlapped at three reference sites (Figure 3), no elk environmental contact behaviors were recorded at these sites, and mule deer were again only observed contacting the environment at one of these sites.

However, network visualizations demonstrate substantial differences in proportions of exposure risk for each species at our three site types (Figure 5A). Most notably, because elk were only observed engaged in environmental contact behaviors at water tanks, all risk was concentrated at these sites. Similarly, mule deer environmental exposure risk occurred predominantly at water tanks (49.1%) and scrape sites (42.7%), with only 8.2% of all risk resulting from contact behaviors at reference sites. Nearly three‐quarters of all white‐tailed deer environmental exposure risk occurred at scrape sites (72.5%), while the remainder was concentrated at reference sites (27.5%). Network graphs also highlighted notable differences in the weights of environmental risk contributed by intra‐ vs. interspecific exposure at each site type. Here, the weight of intraspecific environmental exposure risk for mule deer and white‐tailed deer was greater at scrape than reference sites. Interestingly, intraspecific risk was higher for mule deer than white‐tailed deer at scrape sites and interspecific risk was overall low (Figure 5A). In contrast, the weight of both intra‐ and interspecific risk was comparatively high for both mule deer and elk at water tanks, and especially for elk when assuming equal competence (Figure 5A).

**FIGURE 5:**
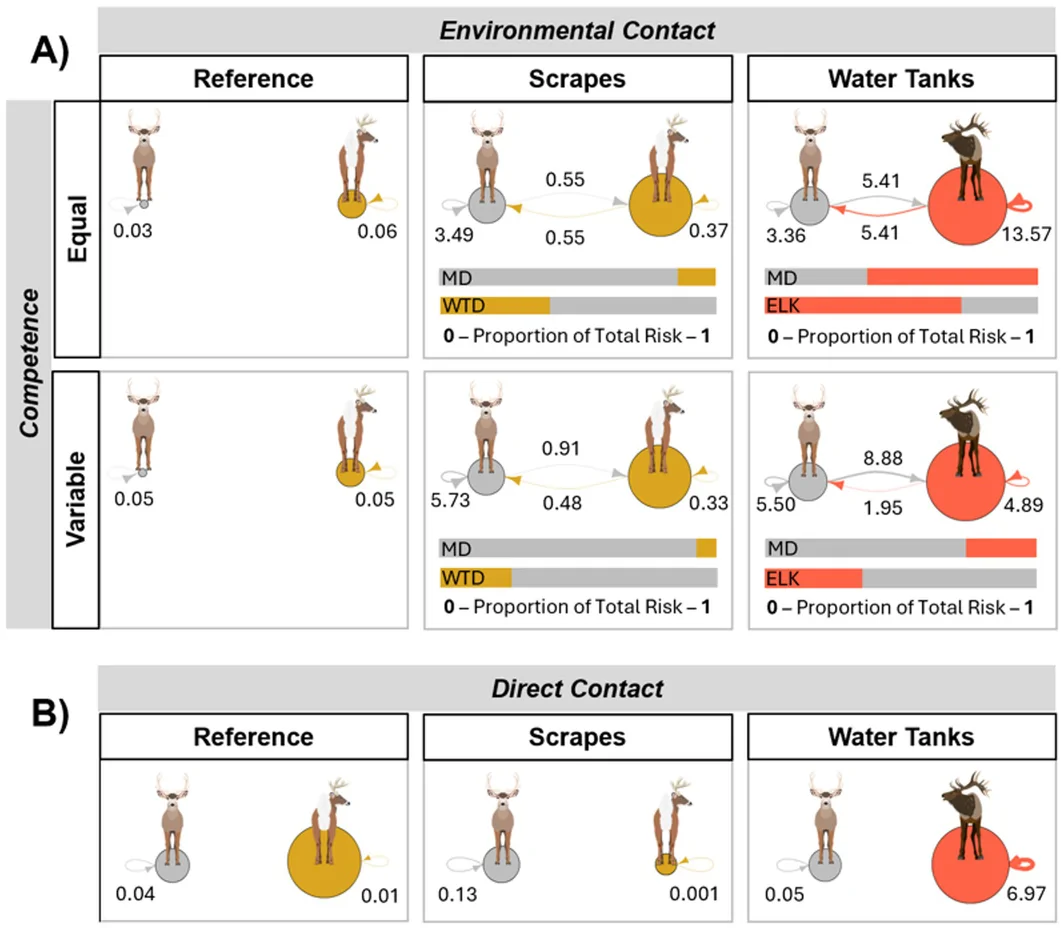
Network visualization of (A) environmental intra‐ and interspecific exposure risk and (B) direct exposure risk at three site types (reference, water tank, scrape). The size of network nodes (circles) represent the proportion of exposure risk to a species (gray = mule deer, yellow = white‐tailed deer, red = elk) across site types. Arrows connecting nodes are directed edges that denote the weight of interspecific exposure risk to a given species from other cervid species at each site type, and arrows looping back on a node are self‐loop edges that denote the weight of intraspecific exposure risk. Edge weights are an estimate of total risk and printed for each edge presented. We used environmental contact networks to compare differences in intra‐ and interspecific exposure risk when considering both equal competence across all species (prevalence = 9.15%) and variable competence assuming differences in CWD prevalence for sympatric mule deer (prevalence = 15%), white‐tailed deer (prevalence = 8%), and elk (prevalence = 3.3%). Bar plots underneath network graphs depict differences in the proportion of total risk attributed to intraspecific (left portion of bar) and interspecific (right portion of bar) exposure for species pairs under considerations of equal and variable competence.

#### 3.2.2 Direct Exposure Risk

Mule deer were the only species for which we were able to test for differences in direct exposure risk across site types and we found no statistical differences in risk across scrape, water tank, and reference sites (Figure 4B, Table 3). However, network graphs portrayed limited differences in the relative amount of direct exposure risk experienced by mule deer; risk to mule deer was two times higher at scrape sites when compared to water tanks and reference sites (Figure 5B). For white‐tailed deer, CWD direct exposure risk was approximately 10 times higher at scrape sites (Figure 5B). All direct exposure risk to elk in network visualizations occurred at water tanks (Figure 5B).

### 3.3 Host Competence

We found notable differences in the proportions of total environmental risk contributed by intra‐ vs. interspecific exposure at water tanks for mule deer and elk, the species we estimated to have the greatest differences in host competence based on our systematic literature review (mule deer = 15%, white‐tailed deer = 8%, elk = 3.3%; see *Methods*). Here, the proportion of total risk attributed to intra‐ and interspecific exposure for mule deer and elk at water tanks portrayed completely different relative exposure risk patterns. Assuming equal competence, water tanks posed high intraspecific exposure risk to elk and interspecific risk to mule deer. Assuming variable competence, water tanks posed high intraspecific risk to mule deer and interspecific risk to elk (Figure 5A). In contrast, accounting for differing competence across species had little effect on the balance of intra‐ and interspecific exposures for mule deer and white‐tailed deer at scrape sites. At these sites, risk to mule deer and white‐tailed deer came predominantly from mule deer (Figure 5A).

## 4 Discussion

Our study assessed environmental and direct CWD exposure risk to male cervids at potential local infectious zones during the mating season and explored changes in exposure risk when considering species differences in host competence. We found intraspecific environmental exposure risk was higher for both mule deer and white‐tailed deer at local infectious zones when compared to reference sites, and that elk only engaged in environmental contact behaviors at water tanks. Additionally, when two cervid species spatially overlapped at a monitored site, both species engaged in environmental contact behaviors exclusively at local infectious zones, concentrating all interspecific exposure risk at these sites. We recorded no instances of interspecific exposure risk at reference sites and elevated intraspecific exposure risk for mule deer and white‐tailed deer at local infectious zones, indicating that these sites are likely to serve as CWD environmental transmission hotspots in multi‐host systems (Lessler et al. 2017). Further, the importance of intraspecific vs. interspecific exposure depended on our assumptions about relative competence for mule deer and elk—the two species documented to have the largest differences in CWD prevalence in areas where all cervids in our study system cooccur (Miller et al. 2000; DeVivo et al. 2017). While these results suggest a role of local infectious zones for environmental transmission, we found less evidence for their potential role in direct CWD transmission. Specifically, mule deer were the only species that engaged in direct contact behaviors at all site types, and direct CWD exposure risk did not differ across site types. Taken together, these findings highlight the potential central role of mule deer in environmental CWD transmission amongst multi‐host cervid communities and their resulting vulnerability to infection, given both their high niche overlap with white‐tailed deer and elk at local infectious zones, and indication of higher prevalence in areas where all three species cooccur.

We investigated water tanks and white‐tailed deer scrape sites as two potential environmental transmission hotspots for male cervids during the mating season. Water tanks concentrated environmental exposure risk for elk and mule deer, with both species spending large amounts of time engaged in environmental contact behaviors at these sites (Figures 2 and 5A). While prions can be detected in water in CWD endemic areas, experimental studies indicate that prion persistence in water is relatively limited (Miles et al. 2011; Nichols et al. 2009). However, water tanks concentrate water resources in a manner that could promote prion infectivity and transmission, as prions can actively bind to tank sediments or metal surfaces (e.g., galvanized steel) (Li et al. 2025; Miles et al. 2011; Pritzkow et al. 2018). Videos from our study captured cervids drinking at the edges of water tanks and contacting the metal lips of tanks between drinking bouts, potentially concentrating prions in these areas.

At scrape sites, the bulk of total exposure risk to mule deer was attributed to exposure from shedding behaviors by other mule deer. This also resulted in increased environmental exposure risk to white‐tailed deer (Figure 4A). Given known differences in the scent‐marking behaviors of male mule deer and white‐tailed deer, we expected interspecific exposure risk between mule deer and white‐tailed deer to occur only briefly during mule deer investigations of white‐tailed deer scrape sites. Instead, we found that mule deer and white‐tailed deer visited scrape sites for similar durations (Figure 2). In interpreting these results, it is important to consider that these two species behave differently at scrape sites, which likely equates to differences in prion shedding. During scraping, white‐tailed deer typically investigate the bare scrape patch of soil (oral‐nasal uptake), paw at the ground (potential shedding via interdigital glands; Ness, Zeng, et al. 2022), interact with specific lick branches (oral‐nasal uptake and shedding via saliva), and urinate (shedding), resulting in high potential for prion uptake and deposition. In contrast, male mule deer primarily contact thrashing bushes with their antlers, which may be contaminated by prions found in antler velvet (Angers et al. 2009) and/or saliva (Henderson et al. 2015; Ness, Jacob, et al. 2022). However, antlers are mostly free of velvet at this time, potentially limiting prion deposition occurring via thrashing behavior. This is also supported by our finding of limited oral‐nasal investigation of these sites. Nevertheless, our finding of high probability of spatial overlap between mule deer and white‐tailed deer at scent‐post sites such as scrapes suggests their importance as potential local infectious zones and warrants further investigation. For example, previous network analysis of male white‐tailed deer demonstrated the potential importance of scrapes and age class in CWD transmission dynamics, where young males (< 2.5 years) had the greatest potential to act as superspreaders due to increased movement across and interaction with scrape sites (Hearst et al. 2023; Huang et al. 2024).

The importance of between‐species environmental exposure risk as a component of total risk depended on the relative CWD competence of sympatric host species. Most notably, if equal host competence is assumed for mule deer and elk, prions deposited into the environment by elk constituted the majority of mule deer environmental exposure risk at water tanks where the two species overlapped. In contrast, when assuming mule deer to be a more competent host than elk (i.e., CWD = 15% and 3.3% in mule deer and elk, respectively; Table 2), most environmental exposure risk to mule deer and elk originated from mule deer (Figure 5A). In contrast, the two‐fold difference in competence between mule deer and white‐tailed deer (15% vs. 8% respectively, Table 2) resulted in only subtle differences in intra‐ vs. interspecific risk compared to the equal host competence scenario (Figure 5A).

Mysterud (2023) recently formalized how these differences in host competence and spatial overlap are likely to affect multi‐host cervid transmission dynamics in areas impacted by CWD by adapting the community epidemiology framework, which aims to describe how disease circulates and emerges in multi‐host communities (Fenton and Pedersen 2005). Our findings align with expectations of the CWD community epidemiological framework, with overall proportions of intra‐ and interspecific environmental risk to mule deer and white‐tailed deer at scrape sites mirroring true‐multi‐host dynamics in both competence scenarios, and risk to mule deer and elk at water tanks resulting in greater risk to elk when considering large differences in host competence (figure 5A; Mysterud 2023).

Here, we emphasize that host CWD competence encompasses a complex interplay of behavioral and individual factors that are presently poorly understood (Barron et al. 2015; Mysterud 2023). The values used in our variable competence scenario serve as a rough approximation given the dearth of studies documenting prevalences in sympatric cervids, highlighting an important area of future research. Interestingly, while some variation in prevalence ratios between species across populations should be expected, a recently published study by Russell et al. (2026) that documents CWD prevalences in mule deer and white‐tailed deer in Wyoming's Wind River Basin found an identical prevalence ratio between mule deer and white‐tailed deer (1.8) as DeVivo et al. 2017 (Table 2). Proportions of CWD cases in mule deer, white‐tailed deer, and elk over 20 years of passive surveillance in Saskatchewan, Canada by the Canadian Wildlife Surveillance Program (Schutten et al. 2026) also reflected similar ratios between mule deer and white‐tailed deer (2.1); however, the ratio between mule deer and elk appears to be lower in this region (2.6). Host competence differences and their resulting impacts on multi‐host transmission dynamics will only be fully elucidated by additional work in this arena, and our analysis provides preliminary insight into how this important dimension of community epidemiology may shape exposure risk.

Finally, we captured a subset of social behaviors that can result in direct CWD exposure risk during the mating season, but these behaviors are likely to vary across species, seasons, and age classes. For example, elk rut earlier than sympatric deer (van Beest et al. 2013), thus our study period primarily captured male elk in post‐rut recovery when bachelor groups might be drawn to water tanks to recuperate. In contrast, mule deer and white‐tailed deer are known to engage in different courtship strategies, with mule deer courting in mixed buck‐doe groups, and white‐tailed deer more commonly engaged in mating activities in isolation (Airst and Lingle 2019). Our results supported these patterns and highlight that transmission via direct contact likely varies both across the span of the mating season and with respect to species‐specific social dynamics. Age class and body size differences are also likely to shape male cervid exposure risk, as not all males engage in rutting activities and behavioral strategies during the mating season (Airst and Lingle 2020).

Our study provides a starting point for understanding how intra‐ and interspecific variation in male cervid spatial overlap, contact behavior, and host competence shape exposure risk at potential local infectious zones during the mating season on a mixed agricultural‐wildland landscape. When considering variable host competence across species, our findings suggest that accounting for interspecific CWD transmission is important for understanding overall exposure risk. For example, in areas were CWD spillover to elk is of concern, mitigating shared use of water tanks with mule deer (e.g., via species‐specific exclusion devices) or transmission potential (e.g., via seasonal disinfection of metal water tank surfaces) could limit the potential for CWD emergence in elk. Similarly, in areas where mule deer populations are declining, better understanding of multi‐host CWD transmission dynamics may prove useful in conserving vulnerable mule deer populations by managing cervid densities, competition, and aggregation points (Cortez and Duffy 2021; Gervasi et al. 2015; Keesing et al. 2006, 2010). This may be particularly important in areas where white‐tailed deer densities and CWD prevalence are high (e.g., Edmunds et al. 2016) and white‐tailed deer could potentially amplify risk to mule deer (Paull et al. 2012; Titcomb et al. 2023). Despite uncertainties surrounding transmission dynamics and host competencies, further refinement and understanding of CWD in a community epidemiological context holds strong promise for more in depth understanding of this how this pervasive disease permeates and persists in cervid populations. Finally, while framed in the context of CWD, the approach used in this study can be applied to any multi‐host system in which differences in host competence are expected for a horizontally transmitted disease.

## Author Contributions

**Rebecca M. Windell:** conceptualization (equal), data curation (lead), formal analysis (equal), funding acquisition (supporting), investigation (lead), methodology (equal), visualization (lead), writing – original draft (lead), writing – review and editing (equal). **Jennifer L. Malmberg:** funding acquisition (equal), investigation (equal), supervision (equal), writing – review and editing (equal). **Larissa L. Bailey:** funding acquisition (equal), supervision (equal), writing – review and editing (equal). **Scott Fesmire:** investigation (equal), writing – review and editing (supporting). **Sydney Simpson:** data curation (equal), writing – review and editing (supporting). **Marnee K. Roundtree:** project administration (supporting), writing – review and editing (supporting). **Georgia C. Titcomb:** conceptualization (equal), formal analysis (supporting), funding acquisition (equal), methodology (equal), supervision (equal), validation (equal), writing – review and editing (equal).

## Funding

This work was supported by the National Wildlife Research Center, 23‐7488‐1759‐CA.

## Conflicts of Interest

The authors declare no conflicts of interest.

## Data Availability

All data, metadata, and R scripts used in this study are archived and publicly available in the following Dryad Digital Repository: https://doi.org/10.5061/dryad.zpc866tpp.

## References

[ece374463-bib-0001] Airst, J. , and S. Lingle . 2019. “Courtship Strategies of White‐Tailed Deer and Mule Deer Males When Living in Sympatry.” Behaviour 156, no. 3–4: 307–330. 10.1163/1568539X-00003543.

[ece374463-bib-0002] Airst, J. I. , and S. Lingle . 2020. “Male Size and Alternative Mating Tactics in White‐Tailed Deer and Mule Deer.” Journal of Mammalogy 101, no. 5: 1231–1243. 10.1093/jmammal/gyaa109.

[ece374463-bib-0003] Almberg, E. S. , P. C. Cross , C. J. Johnson , D. M. Heisey , and B. J. Richards . 2011. “Modeling Routes of Chronic Wasting Disease Transmission: Environmental Prion Persistence Promotes Deer Population Decline and Extinction.” PLoS One 6, no. 5: e19896. 10.1371/journal.pone.0019896. PMC3094393 21603638

[ece374463-bib-0004] Angers, R. C. , T. S. Seward , D. Napier , et al. 2009. “Chronic Wasting Disease Prions in Eik Antler Velvet.” Emerging Infectious Diseases 15, no. 5: 696–703. 10.3201/eid1505.081458. PMC2687044 19402954

[ece374463-bib-0005] Barron, D. G. , S. S. Gervasi , J. N. Pruitt , and L. B. Martin . 2015. “Behavioral Competence: How Host Behaviors Can Interact to Influence Parasite Transmission Risk.” Current Opinion in Behavioral Sciences 6: 35–40. 10.1016/j.cobeha.2015.08.002.

[ece374463-bib-0006] Blackburn, J. K. , H. H. Ganz , J. M. Ponciano , et al. 2019. “Modeling R 0 for Pathogens With Environmental Transmission: Animal Movements, Pathogen Populations, and Local Infectious Zones.” International Journal of Environmental Research and Public Health 16, no. 6: 954. 10.3390/ijerph16060954. PMC6466347 30884913

[ece374463-bib-0007] Brooks, M. E. , K. Kristensen , K. J. van Benthem , A. Magnusson , and C. W. Berg . 2017. “glmmTMB Balances Speed and Flexibility Amongst Packages for glmmTMB Balances Speed and Flexibility Amongst Packages for Zero‐inflated Generalized Linear Mixed Modeling Zero‐inflated Generalized Linear Mixed Modeling.” Part of the Numerical Analysis and Scientific Computing Commons, and the Programming Languages and Compilers Commons. https://digitalcommons.unl.edu/r‐journal.

[ece374463-bib-0008] Cortez, M. H. , and M. A. Duffy . 2021. “The Context‐Dependent Effects of Host Competence, Competition, and Pathogen Transmission Mode on Disease Prevalence.” American Naturalist 198, no. 2: 179–194. 10.1086/715110. 34260871

[ece374463-bib-0009] Craft, M. E. 2015. “Infectious Disease Transmission and Contact Networks in Wildlife and Livestock.” Philosophical Transactions of the Royal Society, B: Biological Sciences 370, no. 1669: 20140107. 10.1098/rstb.2014.0107. PMC4410373 25870393

[ece374463-bib-0010] Csardi, G. 2014. “The igraph Software Package for Complex Network Research.” https://www.researchgate.net/publication/221995787.

[ece374463-bib-0011] De Vos, A. , P. Brokx , and V. Geist . 1967. “A Review of Social Behavior of the North American Cervids During the Reproductive Period.” American Midland Naturalist 77, no. 2: 390.

[ece374463-bib-0012] Denkers, N. D. , E. E. McNulty , C. N. Kraft , et al. 2024. “Temporal Characterization of Prion Shedding in Secreta of White‐Tailed Deer in Longitudinal Study of Chronic Wasting Disease, United States.” Emerging Infectious Diseases 30, no. 10: 2118–2127. 10.3201/eid3010.240159. PMC11431932 39320164

[ece374463-bib-0013] DeVivo, M. T. , D. R. Edmunds , M. J. Kauffman , et al. 2017. “Endemic Chronic Wasting Disease Causes Mule Deer Population Decline in Wyoming.” PLoS One 12, no. 10: e0186512. 10.1371/journal.pone.0186512. PMC5648191 29049389

[ece374463-bib-0014] Dushoff, J. , M. P. Kain , and B. M. Bolker . 2019. “I Can See Clearly Now: Reinterpreting Statistical Significance.” Methods in Ecology and Evolution 10, no. 6: 756–759. 10.1111/2041-210X.13159.

[ece374463-bib-0015] Edmunds, D. R. , M. J. Kauffman , B. A. Schumaker , et al. 2016. “Chronic Wasting Disease Drives Population Decline of White‐Tailed Deer.” PLoS One 11, no. 8: e0161127. 10.1371/journal.pone.0161127. PMC5004924 27575545

[ece374463-bib-0016] Escobar, L. E. , S. Pritzkow , S. N. Winter , et al. 2020. “The Ecology of Chronic Wasting Disease in Wildlife.” Biological Reviews 95, no. 2: 393–408. 10.1111/brv.12568. PMC7085120 31750623

[ece374463-bib-0017] Farine, D. R. , and H. Whitehead . 2015. “Constructing, Conducting and Interpreting Animal Social Network Analysis.” Journal of Animal Ecology 84, no. 5: 1144–1163. 10.1111/1365-2656.12418. PMC4973823 26172345

[ece374463-bib-0018] Fenton, A. , and A. B. Pedersen . 2005. “Community Epidemiology Framework for Classifying Disease Threats.” Emerging Infectious Diseases 11, no. 12: 1815–1821. www.cdc.gov/. 10.3201/eid1112.050306 PMC3367628 16485464

[ece374463-bib-0019] Gervasi, S. S. , D. J. Civitello , H. J. Kilvitis , and L. B. Martin . 2015. “The Context of Host Competence: A Role for Plasticity in Host–Parasite Dynamics.” Trends in Parasitology 31, no. 9: 419–425. 10.1016/j.pt.2015.05.002. PMC4567474 26048486

[ece374463-bib-0020] Greenberg, S. , T. Godin , and J. Whittington . 2019. “Design Patterns for Wildlife‐Related Camera Trap Image Analysis.” Ecology and Evolution 9, no. 24: 13706–13730. 10.1002/ece3.5767. PMC6953665 31938476

[ece374463-bib-0021] Haley, N. J. , K. Merrett , A. B. Stein , et al. 2019. “Estimating Relative CWD Susceptibility and Disease Progression in Farmed White‐Tailed Deer With Rare PRNP Alleles.” PLoS One 14, no. 12: e0224342. 10.1371/journal.pone.0224342. PMC6886763 31790424

[ece374463-bib-0022] Haley, N. J. , R. Rielinger , K. A. Davenport , K. O'Rourke , G. Mitchell , and J. A. Richt . 2017. “Estimating Chronic Wasting Disease Susceptibility in Cervids Using Real‐Time Quaking‐Induced Conversion.” Journal of General Virology 98, no. 11: 2882–2892. 10.1099/jgv.0.000952. PMC5845664 29058651

[ece374463-bib-0023] Hartig, F. 2024. “DHARMa: Residual Diagnostics for Hierarchical (Multi‐Level/Mixed) Regression Models.” (R package version 0.4.7). http://florianhartig.github.io/DHARMa/.

[ece374463-bib-0024] Hearst, S. , M. Huang , B. Johnson , and E. Rummells . 2023. “Identifying Potential Super‐Spreaders and Disease Transmission Hotspots Using White‐Tailed Deer Scraping Networks.” Animals 13, no. 7: 1171. 10.3390/ani13071171. PMC10093032 37048427

[ece374463-bib-0025] Heffelfinger, J. R. , and P. R. Krausman . 2023. Ecology and Management of Black‐Tailed and Mule Deer of North America. CRC Press.

[ece374463-bib-0026] Henderson, D. M. , K. A. Davenport , N. J. Haley , N. D. Denkers , C. K. Mathiason , and E. A. Hoover . 2015. “Quantitative Assessment of Prion Infectivity in Tissues and Body Fluids by Real‐Time Quaking‐Induced Conversion.” Journal of General Virology 96, no. 1: 210–219. 10.1099/vir.0.069906-0. PMC4268821 25304654

[ece374463-bib-0027] Henderson, D. M. , J. M. Tennant , N. J. Haley , N. D. Denkers , C. K. Mathiason , and E. A. Hoover . 2017. “Detection of Chronic Wasting Disease Prion Seeding Activity in Deer and Elk Feces by Real‐Time Quaking‐Induced Conversion.” Journal of General Virology 98, no. 7: 1953–1962. 10.1099/jgv.0.000844. PMC5817259 28703697

[ece374463-bib-0028] Huang, M. H. J. , S. Demarais , B. K. Strickland , A. Houston , A. Banda , and K. C. VerCauteren . 2024. “Chronic Wasting Disease Effects on a Breeding Season Behavior in White‐Tailed Deer ( *Odocoileus virginianus* ).” Journal of Mammalogy 105, no. 5: 1175–1181. 10.1093/jmammal/gyae050. PMC11427538 39345854

[ece374463-bib-0029] Johnson, C. , J. Johnson , J. P. Vanderloo , D. Keane , J. M. Aiken , and D. McKenzie . 2006. “Prion Protein Polymorphisms in White‐Tailed Deer Influence Susceptibility to Chronic Wasting Disease.” Journal of General Virology 87, no. 7: 2109–2114. 10.1099/vir.0.81615-0. 16760415

[ece374463-bib-0030] Johnson, C. J. , A. Herbst , C. Duque‐Velasquez , et al. 2011. “Prion Protein Polymorphisms Affect Chronic Wasting Disease Progression.” PLoS One 6, no. 3: e17450. 10.1371/journal.pone.0017450. PMC3060816 21445256

[ece374463-bib-0031] Keesing, F. , L. K. Belden , P. Daszak , et al. 2010. “Impacts of Biodiversity on the Emergence and Transmission of Infectious Diseases.” Nature 468, no. 7324: 647–652. 10.1038/nature09575. PMC7094913 21124449

[ece374463-bib-0032] Keesing, F. , R. D. Holt , and R. S. Ostfeld . 2006. “Effects of Species Diversity on Disease Risk.” Ecology Letters 9, no. 4: 485–498. 10.1111/j.1461-0248.2006.00885.x. 16623733

[ece374463-bib-0033] Kraft, C. N. , N. D. Denkers , C. K. Mathiason , and E. A. Hoover . 2023. “Longitudinal Detection of Prion Shedding in Nasal Secretions of CWD‐Infected White‐Tailed Deer.” Journal of General Virology 104, no. 1: 001825. 10.1099/jgv.0.001825. PMC10233467 36748533

[ece374463-bib-0034] Lenth, R. V. 2025. “emmeans: Estimated Marginal Means, aka Least‐Squares Means.” (R package version 1.10.7). https://CRAN.R‐project.org/package=emmeans.

[ece374463-bib-0035] Lessler, J. , A. S. Azman , H. S. McKay , and S. M. Moore . 2017. “Perspective Piece: What Is a Hotspot Anyway?” American Journal of Tropical Medicine and Hygiene 96, no. 6: 1270–1273. 10.4269/ajtmh.16-0427.

[ece374463-bib-0036] Li, E. A. , D. L. Karwan , S. S. Lichtenberg , et al. 2025. “Prion Partitioning and Persistence in Environmental Waters.” Environmental Science and Technology 59, no. 11: 5715–5725. 10.1021/acs.est.4c11497. PMC11948337 40080723

[ece374463-bib-0037] Mejía‐Salazar, M. F. , C. L. Waldner , Y. Ten Hwang , and T. K. Bollinger . 2018. “Use of Environmental Sites by Mule Deer: A Proxy for Relative Risk of Chronic Wasting Disease Exposure and Transmission.” Ecosphere 9, no. 1: e02055. 10.1002/ecs2.2055.

[ece374463-bib-0038] Miles, S. L. , K. Takizawa , C. P. Gerba , and I. L. Pepper . 2011. “Survival of Infectious Prions in Water.” Journal of Environmental Science and Health, Part A, Toxic/Hazardous Substances & Environmental Engineering 46, no. 9: 938–943. 10.1080/10934529.2011.586247. 21707419

[ece374463-bib-0039] Miller, M. W. , N. T. Hobbs , and S. J. Tavener . 2006. “Dynamics of Prion Disease Transmission in Mule Deer.” Ecological Applications 16, no. 6: 2208–2214. 10.1890/1051-0761(2006)016[2208:DOPDTI]2.0.CO;2. 17205898

[ece374463-bib-0040] Miller, M. W. , E. S. Williams , C. W. Mccarty , et al. 2000. “Epizootiology of Chronic Wasting Disease in Free‐Ranging Cervids in Colorado and Wyoming.” Journal of Wildlife Diseases 34, no. 4: 676–690. http://meridian.allenpress.com/jwd/article‐pdf/36/4/676/2233787/0090‐3558‐36_4_676.pdf. 10.7589/0090-3558-36.4.676 11085429

[ece374463-bib-0041] Mysterud, A. 2023. “An Epidemiological Framework for Chronic Wasting Disease in Multihost Communities.” Journal of Applied Ecology 60, no. 8: 1545–1550. 10.1111/1365-2664.14441.

[ece374463-bib-0042] Mysterud, A. , E. J. Solberg , E. L. Meisingset , et al. 2023. “Estimating and Managing Broad Risk of Chronic Wasting Disease Spillover Amongst Cervid Species.” Ecosphere 14, no. 9: e4663. 10.1002/ecs2.4663.

[ece374463-bib-0043] Ness, A. , A. Jacob , K. Saboraki , et al. 2022. “Cellular Prion Protein Distribution in the Vomeronasal Organ, Parotid, and Scent Glands of White‐Tailed Deer and Mule Deer.” Prion 16, no. 1: 40–57. 10.1080/19336896.2022.2079888. PMC9154781 35634740

[ece374463-bib-0044] Ness, A. , D. Zeng , A. Kuznetsova , et al. 2022. “Chronic Wasting Disease Prions in Mule Deer Interdigital Glands.” PLoS One 17, no. 10: e0275375. 10.1371/journal.pone.0275375. PMC9529147 36190981

[ece374463-bib-0045] NGPC . 2024. “Chronic Wasting Disease Management Plan.”

[ece374463-bib-0046] Nichols, T. A. , B. Pulford , A. C. Wyckoff , et al. 2009. “Detection of Protease‐Resistant Cervid Prion Protein in Water From a CWD‐Endemic Area.” Prion 3, no. 3: 171–183. 10.4161/pri.3.3.9819. PMC2802782 19823039

[ece374463-bib-0047] Nobert, B. R. , E. H. Merrill , M. J. Pybus , T. K. Bollinger , and Y. T. Hwang . 2016. “Landscape Connectivity Predicts Chronic Wasting Disease Risk in Canada.” Journal of Applied Ecology 53, no. 5: 1450–1459. 10.1111/1365-2664.12677.

[ece374463-bib-0048] Paull, S. H. , S. Song , K. M. McClure , L. C. Sackett , A. M. Kilpatrick , and P. T. J. Johnson . 2012. “From Superspreaders to Disease Hotspots: Linking Transmission Across Hosts and Space.” Frontiers in Ecology and the Environment 10, no. 2: 75–82. 10.1890/110111. PMC3589764 23482675

[ece374463-bib-0049] Plummer, I. H. , S. D. Wright , C. J. Johnson , J. A. Pedersen , and M. D. Samuel . 2017. “Temporal Patterns of Chronic Wasting Disease Prion Excretion in Three Cervid Species.” Journal of General Virology 98, no. 7: 1932–1942. 10.1099/jgv.0.000845. 28708047

[ece374463-bib-0050] Potapov, A. , E. Merrill , M. Pybus , D. Coltman , and M. A. Lewis . 2013. “Chronic Wasting Disease: Possible Transmission Mechanisms in Deer.” Ecological Modelling 250: 244–257. 10.1016/j.ecolmodel.2012.11.012.

[ece374463-bib-0051] Potapov, A. , E. Merrill , M. Pybus , and M. A. Lewis . 2016. “Chronic Wasting Disease: Transmission Mechanisms and the Possibility of Harvest Management.” PLoS One 11, no. 3: e0151039. 10.1371/journal.pone.0151039. PMC4786122 26963921

[ece374463-bib-0052] Pritzkow, S. 2022. “Transmission, Strain Diversity, and Zoonotic Potential of Chronic Wasting Disease.” Viruses 14, no. 7: 1390. 10.3390/v14071390. PMC9316268 35891371

[ece374463-bib-0053] Pritzkow, S. , R. Morales , A. Lyon , L. Concha‐Marambio , A. Urayama , and C. Soto . 2018. “Efficient Prion Disease Transmission Through Common Environmental Materials.” Journal of Biological Chemistry 293, no. 9: 3363–3373. 10.1074/jbc.M117.810747. PMC5836136 29330304

[ece374463-bib-0054] R Core Team . 2024. R: A Language and Environment for Statistical Computing. R Foundation for Statistical Computing. https://www.R‐project.org/.

[ece374463-bib-0055] Rees, E. E. , E. H. Merrill , T. K. Bollinger , Y. Ten Hwang , M. J. Pybus , and D. W. Coltman . 2012. “Targeting the Detection of Chronic Wasting Disease Using the Hunter Harvest During Early Phases of an Outbreak in Saskatchewan, Canada.” Preventive Veterinary Medicine 104, no. 1–2: 149–159. 10.1016/j.prevetmed.2011.10.016. 22137503

[ece374463-bib-0056] Rogers, W. , E. E. Brandell , and P. C. Cross . 2022. “Epidemiological Differences Between Sexes Affect Management Efficacy in Simulated Chronic Wasting Disease Systems.” Journal of Applied Ecology 59, no. 4: 1122–1133. 10.1111/1365-2664.14125.

[ece374463-bib-0057] Russell, T. I. , P. C. Cross , B. S. Dugovich , et al. 2026. “Chronic Wasting Disease Reduces Mule Deer Survival and Hunter Success in the Wind River Basin of Wyoming.” From the Field 1, no. 1: 41–60.

[ece374463-bib-0058] Sandoval, A. M. , A. V. Nalls , E. E. McNulty , et al. 2025. “Vertical Transmission of Chronic Wasting Disease in Free‐Ranging White‐Tailed Deer Populations.” Scientific Reports 15, no. 1: 28553. 10.1038/s41598-025-12727-8. PMC12325800 40764633

[ece374463-bib-0059] Saunders, S. E. , S. L. Bartelt‐Hunt , and J. C. Bartz . 2012. “Occurrence, Transmission, and Zoonotic Potential of Chronic Wasting Disease.” Emerging Infectious Diseases 18, no. 3: 369–376. 10.3201/eid1803.110685. PMC3309570 22377159

[ece374463-bib-0060] Saunders, S. E. , J. C. Bartz , and S. L. Bartelt‐Hunt . 2012. “Soil‐Mediated Prion Transmission: Is Local Soil‐Type a Key Determinant of Prion Disease Incidence?” Chemosphere 87, no. 7: 661–667. 10.1016/j.chemosphere.2011.12.076. 22265680

[ece374463-bib-0061] Schauber, E. M. , C. K. Nielsen , L. J. Kjær , C. W. Anderson , and D. J. Storm . 2015. “Social Affiliation and Contact Patterns Amongst White‐Tailed Deer in Disparate Landscapes: Implications for Disease Transmission.” Journal of Mammalogy 96, no. 1: 16–28. 10.1093/jmammal/gyu027. PMC4668922 26937044

[ece374463-bib-0062] Schutten, K. , L. Shirose , B. Stevens , et al. 2026. “Twenty Years of Ungulate Disease Surveillance by the Canadian Wildlife Health Cooperative (2003–2022).” PLoS One 21, no. 3: e0343520. 10.1371/journal.pone.0343520. PMC12962481 41785212

[ece374463-bib-0063] Smolko, P. , D. Seidel , M. Pybus , A. Hubbs , M. Ball , and E. Merrill . 2021. “Spatio‐Temporal Changes in Chronic Wasting Disease Risk in Wild Deer During 14 Years of Surveillance in Alberta, Canada.” Preventive Veterinary Medicine 197: 105512. 10.1016/j.prevetmed.2021.105512. 34740023

[ece374463-bib-0064] Storm, D. J. , M. D. Samuel , R. E. Rolley , et al. 2013. “Deer Density and Disease Prevalence Influence Transmission of Chronic Wasting Disease in White‐Tailed Deer.” Ecosphere 4, no. 1: 1–14. 10.1890/ES12-00141.1.

[ece374463-bib-0065] Taylor, L. H. , S. M. Latham , and M. E. J. Woolhouse . 2001. “Risk Factors for Human Disease Emergence.” Philosophical Transactions of the Royal Society, B: Biological Sciences 356, no. 1411: 983–989. 10.1098/rstb.2001.0888. PMC1088493 11516376

[ece374463-bib-0066] Titcomb, G. , J. Hulke , J. N. Mantas , B. Gituku , and H. Young . 2023. “Cattle Aggregations at Shared Resources Create Potential Parasite Exposure Hotspots for Wildlife.” Proceedings of the Royal Society B: Biological Sciences 290, no. 2012: 20232239. 10.1098/rspb.2023.2239. PMC10697799 38052242

[ece374463-bib-0067] Titcomb, G. , J. N. Mantas , J. Hulke , I. Rodriguez , D. Branch , and H. Young . 2021. “Water Sources Aggregate Parasites With Increasing Effects in More Arid Conditions.” Nature Communications 12, no. 1: 7066. 10.1038/s41467-021-27352-y. PMC8642388 34862389

[ece374463-bib-0068] van Beest, F. M. , E. V. Wal , A. V. Stronen , and R. K. Brook . 2013. “Factors Driving Variation in Movement Rate and Seasonality of Sympatric Ungulates.” Journal of Mammalogy 94, no. 3: 691–701. 10.1644/12-MAMM-A-080.1.

[ece374463-bib-0069] Venables, W. N. , and B. D. Ripley . 2002. Modern Applied Statistics With S. 4th ed. Springer.

[ece374463-bib-0071] Zabel, M. , and A. Ortega . 2017. “The Ecology of Prions.” Microbiology and Molecular Biology Reviews 81, no. 3: e00001–17. 10.1128/mmbr.00001-17. PMC5584314 28566466

